# Comparing the Quality and Complications of Tube Thoracostomy by Emergency Medicine and Surgery Residents; a Cohort Study

**Published:** 2017-01-11

**Authors:** Parvin Kashani, Sepideh Harati, Ali Shirafkan, Alireza Amirbeigi, Hamid Reza Hatamabadi

**Affiliations:** 1 Emergency Department, Loghman Hakim Hospital, Shahid Beheshti University of Medical Sciences, Tehran, Iran.; 2 Emergency Department, Imam Hossein Hospital, Shahid Beheshti University of Medical Sciences, Tehran, Iran.; 3 Surgery Department, University of Texas Medical Branch, Galveston, Texas, USA.; 4 Surgery Department, Imam Hossein Hospital, Shahid Beheshti University of Medical Sciences, Tehran, Iran.; 5 Sina Trauma and Surgery Research Center, Tehran University of Medical Sciences, Tehran, Iran.

**Keywords:** Thoracostomy, emergency medicine, general surgery, postoperative complications

## Abstract

**Introduction:**

Tube thoracostomy complications depend on the operator’s skill, patients’ general condition and the place in which the procedure is done. The present study aimed to compare the quality and complications of tube thoracostomy carried out by emergency medicine residents (EMRs) and surgery residents (SRs).

**Methods:**

This cohort study was conducted on 18-60 years old trauma patients in need of tube thoracostomy presenting to two academic emergency departments. Quality of tube placement and its subsequent complications until tube removal were compared between SRs and EMRs using SPSS 20.

**Results:**

72 patients with the mean age of 37.1 ± 14.1 years were studied (86.1% male). 23 (63.8%) cases were complicated in SRs and 22 (61.1%) cases in EMRs group (total= 62.5%). Chest drain dislodgement (22.2% in SRs vs. 22.2% EMRs; p>0.99), drainage failure (19.4% in SRs vs. 16.7% EMRs; p=0.50), and surgical site infection (11.1% in SRs vs. 19.4% EMRs; p=0.25) were among the most common observed complications. The overall odds ratio of complication development was 0.89 (95% CI: 0.35-2.25, p = 0.814) for SRs and 1.12 (95% CI: 0.28-4.53, p = 0.867) for EMRs.

**Conclusion:**

The findings of the present study showed no significant difference between SRs and EMRs regarding quality of tube thoracostomy placement and its subsequent complications for trauma patients. The rate of complications were interestingly high (>60%) for both groups.

## Introduction

Tube Thoracostomy is one of the most frequent life-saving interventions in management of trauma patients (1-3). The necessity of tube thoracostomy for traumatic chest injuries has been questioned due to high complication rate reported by some studies (4, 5). Tube thoracostomy has the potential to cause complications related to insertion, position and infection. These complication rates and types may differ depending on various factors including those related to the patient and the physician (6, 7).

Tube thoracostomy complications largely depend on the knowledge and skill of the operator in addition to the patients’ general condition and the place in which the procedure is done (8). This procedure has traditionally been performed only by thoracic surgeons and surgery residents (SRs). However, in recent years, tube thoracostomy has been performed by other specialties such as emergency medicine residents (EMRs) (9-11). 

Ball et al. estimated the prevalence of complications of thoracostomy to be about 13% when done by SRs and 40% when done by EMRs (12).

In Iranian emergency medicine curriculum, tube thoracostomy training should be provided for all EMRs. The present study aimed to compare the quality and complications of tube thoracostomy between EMRs and SRs of two academic emergency departments.

## Methods

***Study design and setting***

The present cohort study was carried out on trauma patients presenting to the emergency departments of Shoahadaye Hafte-tir and Imam Hossein Hospitals, Tehran, Iran, who underwent tube thoracostomy either by SRs or EMRs. The protocol of the study was approved by the ethics committee of Shahid Beheshti University of medical Sciences. Before entering the study, informed written consent was obtained from the patient or their relative. Throughout the study, researchers adhered to the principles of Helsinki Declaration.

***Participants***

Study population consisted of 18-60 years old multiple trauma patients who had indication for tube thoracostomy according to the current Advanced Traumatic Life Supports (ATLS) protocol. Tube thoracostomy performed in pre-hospital settings or other centres, patients under 18 or over 60 years old, death for any reason other than those related to thoracostomy, and tube thoracostomy under close observation of attending were considered as exclusion criteria in this study. Sampling was performed using convenience method.

***Data gathering***

Using a predesigned checklist, patients’ demographic data (age, sex), vital signs (pulse rate, respiratory rate, blood pressure, O2 saturation), indication of tube thoracostomy (pneumothorax, hemothorax, or hemopneumothorax), and quality of tube placement (abutment to mediastinum, extending caudal from insertion site, and intra-fissure/intra-abdominal/trans-diaphragmatic placement), as well as complications ( chest drain dislodgement, haemorrhage and vascular injury, empyema and surgical site infection, air leak and subcutaneous emphysema, pulmonary laceration or puncture, and drainage failure) were gathered by one observer resident in each hospital. The Observer resident did not interfere with the performed procedure in any stage and only filled out the prepared checklists. 

The procedures had been performed by third level SRs in Imam Hossein Hospital and by third level EMRs in Hafte-tir Hospital. Hafte-tir Hospital (South Tehran) accepts about 250 traumatic patients a day and third level EMRs do all the procedures for trauma patients in emergency department but in Imam Hossein Hospital all chest tubes are inserted by SRs.

After tube thoracostomy, an anterior-posterior chest radiography was performed for all patients and was scored (0 – 5 based on Likert scale) regarding the quality of tube placement by two separate radiologists. If any difference was seen in the two reports, a third radiologist was invited. None of radiologists were informed about the patients group. 

**Outcome**

The occurrence of one of the above-mentioned complications was considered as primary outcome and quality of tube placement as secondary outcome. All patients were followed until tube thoracostomy removal. 

**Statistical analysis**

Based on the study by Ball et al. the prevalence of complications after tube thoracostomy in SRs and EMRs groups were 13% and 40%, respectively (12). Therefore, considering 95% confidence interval (α=0.05) and 80% power, the minimum sample size required for each group was calculated to be 32 cases.

Collected data were were analysed using SPSS software version 20. Chi-square test, t-test and fisher-exact test were used for comparing variables between the two groups. With the aim of eliminating probable bias, a backward multifactorial regression logistic model was designed to show the independent effect of residency on the mentioned complications. Odds ratios were reported with 95% confidence interval (CI). P-value less than 0.05 was considered as significant.

## Results

***Baseline variables***

72 patients with the mean age of 37.1 ± 14.1 years were studied (86.1% male). Baseline characteristics of participants are summarized in table 1. There was no significant difference between the groups regarding patients’ baseline variables. 

***Outcomes***

23 (63.8%) cases of tube thoracostomy were complicated in SRs and 22 (61.1%) cases in EMRs (total= 62.5%). Frequency of tube thoracostomy-related complications in each group is presented in table 2. Chest drain dislodgement (22.2% in SRs vs. 22.2% EMRs; p>0.99), drainage failure (19.4% in SRs vs. 16.7% EMRs; p=0.50), and surgical site infection (11.1% in SRs vs. 19.4% EMRs; p=0.25) were among the most common observed complications. The Odds ratios for developing tube thoracostomy-related complications are presented in table 3. The overall odds ratio of complication developing was 0.89 (95% CI: 0.35-2.25, p = 0.814) for SRs and 1.12 (95% CI: 0.28-4.53, p = 0.867) for EMRs.

Quality of tube placement was not different between SRs and EMRs according to radiologist reports (2.5 ± 1.5 versus 2.1 ± 1.3 score, respectively; p=0.19). 

## Discussion

The findings of the present study showed no significant difference between SRs and EMRs regarding quality of tube thoracostomy placement and its subsequent complications for trauma patients.

The prevalence of tube thoracostomy complications were 63.8% and 61.1% in SRs and EMRs, respectively (total= 62.5%). 

The rate of intercostal artery injury, pleural cavity infection, and retroperitoneal placement of tube thoracostomy was reported to be 37% in Sethuraman et al. study. They revealed that, tube thoracostomy by EMRs has similar complication rates as the other residents and they are generally minor types of complications (13). Aziz et al., in a year-long study in 2010, showed that operator’s skill is one of the most important factors affecting the outcome and complications of tube thoracostomy placement. The complication rates were exceptionally higher if residents did the procedure compared to the specialists. They reported the 36.7% total complication rate consisted of 26.7% technical and 10% infective (14). Total complication rates in the present study were higher than others reports (about 2 times). This higher prevalence rate might be due to lack of knowledge, proper attitude or skills among the studied residents regarding tube thoracostomy. Therefore, holding continuous training courses and performing the procedure under supervision of a specialist may be effective in this regard.

**Table 1:** Baseline characteristics of patients in surgery residents (SR) and emergency medicine residents (EMR) groups

**Variable**	**SR (n=36)**	**EMR (n=36)**	**p**
**Age (year)**	35.9±13.8	38.4±14.8	0.46
**Gender **			
Male	31 (86.1)	33 (91.7)	0.71
Female	5 (13.9)	3 (8.3)	
**Tube thoracostomy indications **			
Non-tension pneumothorax	8 (22.2)	13 (36.1)	0.10
Hemothorax	15 (41.7)	11 (30.5)	0.63
Hemopneumothorax	8 (22.2)	10 (27.8)	0.24
Tension pneumothorax	5 (13.8)	2 (5.5)	>0.99
**Accompanying injury **			
Brain	6(16.7)	4(11.1)	0.50
Spine	4(11.1)	3(8.3)	>0.99
Neck	2(5.6)	3(8.3)	>0.99
Chest	10(27.8)	10(27.8)	>0.99
Pelvic	9(25)	6(16.7)	0.38
Upper limb	7 (19.4)	7(19.4)	>0.99
Lower limb	12(33.3)	5(13.9)	0.05
**Blood pressure ** **(mmHg)**			
Systolic	109.1±18.1	115 ± 18.2	0. 18
Diastolic	67.4±8.8	71.1±12.3	0.15
**Pulse Rate ** **(beat/minute)**	95.3±12.8	94.0±13.5	0.67
**Respiratory Rate ** **(n/minute)**	19.9±3.1	19.3±3.2	0.46
**O2 saturation**	88.7 ± 3.6	89.2 ± 3.6	0.58

Data are presented as mean ± standard deviation or frequency and percentage.

**Table 2:** Frequency of tube thoracostomy complications among surgery residents (SR) and emergency medicine residents (EMR

**Complication**	**SR (n=36)**	**EMR (n=36)**	**p**
**Surgical site** ** infection**	4 (11.1)	7 (19.4)	0.25
**Haemorrhage** ^1^	2 (5.6)	0 (0.0)	0.24
**Pulmonary laceration**	1 (2.8)	1 (2.8)	0.75
**Drain dislodgement**	8 (22.2)	8 (22.2)	>0.99
**Subcutaneous emphysema** ^ 2^	1 (2.8)	0 (0.0)	>0.50
**Drainage Failure **	7 (19.4)	6(16.7)	0.50

Data were presented as number and percentage.1: due to vascular injury, 2: air leak.

**Table 3:** The odds ratio for developing tube thoracostomy-related complications among surgery residents (SR) and emergency medicine residents (EMR

Complication	Odds ratio (95% CI)*	P value
Overall		
**SR** **EMR**	0.89 (0.35-2.25) 1.12 (0.28-4.53)	0.814 0.867
Surgical site infection		
**SR** **EMR**	0.52 (0.14-1.95) 0.65 (0.12-3.63)	0.311 0.621
Pulmonary laceration		
**SR** **EMR**	1.00 (0.60-16.62) 0.00	1.000 0.998
Drain dislodgement		
**SR** **EMR**	1.00 (0.33-3.04) 1.10 (0.24-5.04)	1.000 0.895
Drainage failure		
**SR** **EMR**	1.20 (0.36-4.02) 2.23 (0.47-10.61)	0.760 0.310

* : Not applicable for haemorrhage and subcutaneous emphysema; CI: confidence interval.

Chan et al. did a retrospective study in 1997 and revealed that tube thoracostomy placement in the emergency department does not lead to a higher rate of complication compared to tube insertion in the operating room (15). Ball et al. also did a retrospective study and revealed 88% technical and 12% infective complications. Additionally, tube placements by non-surgery residents outside the trauma wards were the independent prognostic factors for occurrence of complications. 13% of the tubes placed by SRs and 40% of the tubes placed by EMRs had complications (12). 

It is strongly suggested to enhance the quality of education starting with a KAP study (knowledge, attitude, and practice) on SRs and EMRs regarding placement of tube thoracostomy. 

***Limitations***

Carrying out the study in 2 separate centres, and therefore having different conditions in the emergency departments regarding staff, patients and environment, might have prevented fully considering/adhering to characteristics of a cohort among participants, especially regarding selection bias. In addition, not blinding the observer residents might lead to observer bias. Among other limitations of this study is its small sample size.

## Conclusion

The findings of the present study showed no significant difference between SRs and EMRs regarding quality of tube thoracostomy placement and its subsequent complications for trauma patients. The rate of complications were interestingly high (>60%) for both groups.
